# Nine Months' Work and Progress

**Published:** 1918-11-02

**Authors:** 


### THE LEICESTER ROYAL INFIRMARY.

### Nine Months' Work and Progress.

THE report of the work of the past nine months submitted to the quarterly meeting of the Governors of the Leicester Royal Infirmary is one of continued usefulness and service. The civilian admissions numbered 2,851, as compared with 2,545 in the preceding period. The military admissions were 304, as compared with 647. The daily average number of beds occupied was 295, as compared with 314. The number of out-patients showed a very considerable advance on the previous year—viz. 22,033 attendances, against 14,455.

### THE NEED OF ORTHOPÆDIC TREATMENT.

In the orthopædic department the number of patients attended advanced to 10,205. Particular attention is drawn to the fact that for many weeks past the average attendance in this department has been between 400 and 500, 120 to 150 of which were attendances made by discharged soldiers sent by the Local Pensions Committees. At no time except the present would it be right to treat these large numbers in so small a department, and if the matter had been left in the hands of the board, a new department would have been provided. Although hampered and restricted by Government Departments, the question of orthopædic treatment has been daily increasing in importance, but practically nothing had been done in Leicester to make provision for discharged soldiers, except in the small department which the board had organised and fitted up under Dr. Astley Clarke's supervision.

Negotiations are still taking place for the provision of further temporary accommodation, but as the Governors have offered to provide a building and to keep it at the disposal of the Ministry for a period of three years after the termination of the war, it is felt that what is wanted is a permanent and not a temporary building, and only the necessity of consulting Government Departments had prevented the board from meeting the requirements of Dr. Astley Clarke.

Tribute is paid in the report to the work of the honorary and resident medical staff, to the matron, assistant matron, and the nursing staff, and the board indicated that it was their desire to maintain a rate of remuneration for the nursing staff equal to that of a modern up-to-date hospital.

### INCREASING SUBSCRIPTION LIST.

The ordinary income for the nine months was £30,818, and the ordinary expenditure £34,102. Legacies had been received to the total value of £5,600, and had been capitalised mostly in the purchase of property to extend the ground area of the institution. Satisfaction was expressed at the continued success attending Mr. Alfred Corah's effort to increase the annual subscription list; £8,000 had been raised as compared with £7,000 last year. The board reported with gratification several additions to the number of endowed beds and cots. Sir Edward Wood, by his will, had endowed two beds in the new wing, and a cot in the children's hospital. The executors of the late Mr. Stanley Broadbent had endowed a cot to his memory. An anonymous friend and his wife had endowed a cot to the memory of their daughter. Mr. Ezra Pickard, with 500 War Savings Certificates, had endowed a third cot, and the Leicester Amateur Music and Dramatic Society had, by a grant of £118, completed the endowment of their second cot.

A special page of the report is devoted to parades, *fêtes*, etc., and attention is drawn to the fact that since the beginning of the year the total sum received from efforts of this kind had reached the large sum of £1,500, a return which reflected credit on the organisers and shows the keenness displayed in the work of the infirmary.

The report continues that difficulties of supplies had been greater than at any other period of the war, and in the matter of its principal provisions a more or less hand-to-mouth existence was evident. Instances are given of high prices, and fear was expressed that there was little likelihood either of the restrictions being removed or prices being diminished for many months to come. Notwithstanding these and other difficulties, the Governors have in constant view the necessity of increasing the treatment accommodation to cope with after-war needs. With the accomplishment of certain additions, no institution would be better able to make an important treatment centre than the infirmary. It is pointed out that those hospitals with adequate and efficient means and methods of treatment cannot fail to attract the largest share of the money forthcoming for the reconstruction of the health of the nation.

### A HERO'S BEQUEST.

The will of a soldier in the Leicestershire Regiment was recently found upon the battlefield, and among other bequests in a small estate of less than £100 was one of £10 to the infirmary. No attempt would be made to "make capital" out of the incident, but a well-deserved tribute was paid to a hero's generosity, and Leicester residents were asked to keep the incident before them.

In supporting the adoption of the report, the Chairman of the Governors and the High Sheriff of Leicestershire, Mr. T. Fielding Johnson, jun., recorded that it was an "ill wind that blows nobody any good," for while they had to lament the dilatory action of Government Departments in connection with the orthopædic department they had secured a valuable addition to the site area which would be of great importance in future extensions at the infirmary. High tribute was paid to the work of the House Governor and Secretary.

